# Impact of gestational diabetes mellitus diagnosed during the third trimester on pregnancy outcomes: a case-control study

**DOI:** 10.1186/s12884-021-03730-8

**Published:** 2021-03-24

**Authors:** Ryosuke Shindo, Shigeru Aoki, Sayuri Nakanishi, Toshihiro Misumi, Etsuko Miyagi

**Affiliations:** 1 Perinatal Center for Maternity and Neonates, Yokohama City University Medical Center, 4-57 Urafunecho, Minami-ku, Yokohama City, Kanagawa 232-0024 Japan; 2 Department of Biostatistics, Yokohama City University Graduate School of Medicine, Yokohama, Japan; 3 Department of Obstetrics and Gynecology, Yokohama City University School of Medicine, Yokohama, Japan

**Keywords:** Gestational diabetes mellitus, International Association of Diabetes and Pregnancy Study Group, Third trimester, Late pregnancy, Caesarean delivery, Japan

## Abstract

**Background:**

In 2010, the International Association of Diabetes and Pregnancy Study Group (IADPSG) proposed new criteria indicating that gestational diabetes mellitus (GDM) can be diagnosed if the fasting threshold of ≤92 mg/dL, 1-h threshold of ≤180 mg/dL, or 2-h threshold of ≤153 mg/dL are exceeded during the 75-g 2-h oral glucose tolerance test (OGTT) performed at 24–28 weeks of gestation. The World Health Organization (WHO) recommends using the proposed diagnostic threshold values of the IADPSG to diagnose GDM; however, it does not limit the timing of the 75-g OGTT. Since 2010 in Japan, GDM has been diagnosed using the same criteria as that proposed by the WHO. However, neither the JSOG nor the WHO has provided any evidence that it is appropriate to use a threshold beyond the range recommended by the IADPSG.

**Methods:**

This was a single-centre retrospective study based on the medical records and delivery registry database of our centre. We included women who underwent a 50-g glucose challenge test (GCT) with results < 140 mg/dL at 24–28 weeks of gestation and subsequently underwent a 75-g OGTT after 29 weeks of gestation with abnormal glucose tolerance suspected based on clinical findings. The reference values for the 75-g OGTT followed the IADPSG criteria. Subjects were classified into the normal glucose tolerance (NGT) group and the GDM group. The type of delivery and neonatal outcomes of the two groups were compared. A multivariable analysis was performed to match the backgrounds of both groups.

**Results:**

In total, the NGT and GDM group comprised 189 and 49 women, respectively. Emergency caesarean delivery rates were similar in the GDM and NGT groups (10.6 and 12.2%, respectively; adjusted odds ratio [OR], 1.25; 95% confidence interval [CI], 0.43–3.64; *p* = 0.74); however, the elective caesarean delivery rate was higher in the GDM group than in the NGT group (16.3 and 5.3%, respectively, adjusted OR, 3.60; 95% CI, 1.27–10.19; *p* = 0.01). No significant differences were observed in other maternal and neonatal outcomes between both groups.

**Conclusion:**

Although a diagnosis of GDM during the third trimester does not improve pregnancy outcomes, it increases the elective caesarean delivery rate.

## Background

Gestational diabetes mellitus (GDM) is associated with pregnancy and neonatal outcomes. Pregnant women with GDM are at a higher risk of having infants with large-for-gestational-age (LGA) status [1–3], preeclampsia [4, 5], shoulder dystocia [6–8], and neonatal morbidities [9], such as hypoglycaemia, hyperbilirubinemia, and respiratory distress syndrome (RDS) [10]. Furthermore, diagnosing and treating GDM contribute to improved delivery and neonatal outcomes [3, 4, 11]. The Hyperglycemia and Adverse Pregnancy Outcome (HAPO) study [2] showed that there was a linear relationship and no threshold for the association between maternal hyperglycaemia and adverse perinatal events. In 2010, the International Association of Diabetes and Pregnancy Study Group (IADPSG) proposed a new diagnostic criterion for GDM based on the results of the HAPO study: GDM can be diagnosed if the fasting threshold of ≤92 mg/dL, 1-h threshold of ≤180 mg/dL, or 2-h threshold of ≤153 mg/dL are exceeded during the 75-g 2-h oral glucose tolerance test (OGTT) performed at 24–28 weeks of gestation [12]. The World Health Organization (WHO) recommends using the proposed diagnostic threshold values of the IADPSG to diagnose GDM; however, it does not limit the timing of the 75-g OGTT [13]. In 2010, The Japan Society of Obstetrics and Gynaecology (JSOG) proposed that GDM be diagnosed using the thresholds recommended by the IADPSG without limiting the timing of the 75-g OGTT, similar to the WHO. However, neither the JSOG nor the WHO has provided any evidence that it is appropriate to use a threshold beyond the range recommended by the IADPSG. Therefore, this study aimed to determine the impact of diagnosing and treating GDM using the 75-g OGTT performed after 29 weeks of gestation, which is beyond the period recommended by the IADPSG, on delivery and neonatal outcomes.

## Methods

### Research design and subject selection

This was a single-centre, retrospective study based on the medical records and delivery registry database of our centre. This study was conducted with the approval of the Ethics Committee of Yokohama City University. Among women who delivered at our hospital between January 1, 2011 and December 31, 2019, we included those who underwent the 50-g glucose challenge test (GCT) at 24–28 weeks of gestation with results < 140 mg/dL and subsequently underwent the 75-g OGTT after 29 weeks of gestation because of suspected abnormal glucose tolerance based on clinical findings, such as LGA, suspected macrosomia, polyhydramnios, and positive urine sugar levels. Women with multiple gestations were excluded. Similarly, women who had undergone previous caesarean deliveries were excluded because a previous caesarean delivery is a major factor determining future delivery procedures. Reference values for the 75-g OGTT were 92 mg/dL (fasting value), 180 mg/dL (1-h value), and 153 mg/dL (2-h value), as recommended by the IADPSG. GDM was diagnosed if any one of these values was exceeded. Subjects were classified into the normal glucose tolerance (NGT) group and the GDM group, and delivery and neonatal outcomes of these two groups were compared.

### GDM treatment

When GDM was diagnosed, dietary therapy and blood glucose level monitoring using regular glycated haemoglobin (HbA1c) measurements or blood glucose measurements before and 2 h after meals were introduced. HbA1c target levels were < 6.2%, and blood glucose target levels were < 100 mg/dL before meals and < 120 mg/dL 2 h after meals. Insulin therapy was introduced if the target blood glucose levels could not be achieved with dietary therapy.

### Characteristics and outcomes

The following maternal characteristics were collected: age at delivery; height (cm); pre-pregnancy weight (kg); pre-pregnancy body mass index (BMI; kg/m^2^); weight at delivery (kg); BMI at delivery (kg/m^2^); gestational weight gain (kg); and number of previous deliveries. The main outcome was diabetes-related complications, which were defined as any one of the following: macrosomia, shoulder dystocia, neonatal hypoglycaemia, neonatal hyperbilirubinemia, or RDS. Other maternal outcomes were preeclampsia, initiation of insulin, caesarean delivery (overall), elective caesarean delivery, emergency caesarean delivery, and instrumental delivery. Secondary neonatal outcomes were LGA status, small for gestational age (SGA) status, low umbilical artery pH (UApH), low Apgar score (APS), neonatal intensive care unit (NICU) admission, birth weight (g), and gestational age (weeks).

### Term definitions

A birth weight of 4000 g or more was defined as macrosomia. Shoulder dystocia was defined when the shoulder girdle could not be delivered spontaneously after delivery of the infant’s head, and if the McRoberts position, suprapubic compressions, or vaginal manipulation were required. LGA status and SGA status were defined as being above the 90th percentile and being below the 10th percentile, respectively, according to the standard Japanese birth weight chart. Low UApH was defined as < 7.1, and low APS was defined as < 7 at 5 min. Preeclampsia was defined as hypertension (systolic blood pressure ≥ 140 mmHg or diastolic blood pressure ≥ 90 mmHg) accompanied by proteinuria (0.3 g/gCr) that first appeared after 20 weeks of gestation and up to 12 weeks postpartum. Emergency caesarean delivery was defined as an attempted vaginal delivery resulting in the requirement for caesarean delivery for any reason during the progression of labour; it did not include cases of scheduled caesarean delivery. Neonatal hypoglycaemia was defined as blood glucose levels < 50 mg/dL for normal term infants and < 40 mg/dL for preterm infants. Neonatal hyperbilirubinaemia was assumed to require phototherapy for the child. RDS was diagnosed by a neonatologist using chest radiographs or microbubble tests and required the administration of a surfactant.

### Statistical analysis

We used JMP PRO version 15 (SAS Institute Inc., Cary, NC) for statistical analyses. Data are presented as frequency or median and interquartile range (IQR). Comparisons of binary variables were performed with the chi-square test. Kruskal–Wallis test was performed to compare continuous variables; *p* < 0.05 was considered statistically significantly different. Multivariable analysis was performed to adjust for maternal backgrounds, and logistic regression analysis was performed for binary variables. Multiple regression analysis was performed for continuous variables. The adjusted odds ratio (aOR) or adjusted regression coefficient (aRC) and 95% confidence interval (CI) of the GDM group were calculated using the NGT group as a reference with adjustment for confounding factors, such as maternal age, non-pregnant BMI, childbirth experience, and sex of the infant.

## Results

**Fig. 1:**
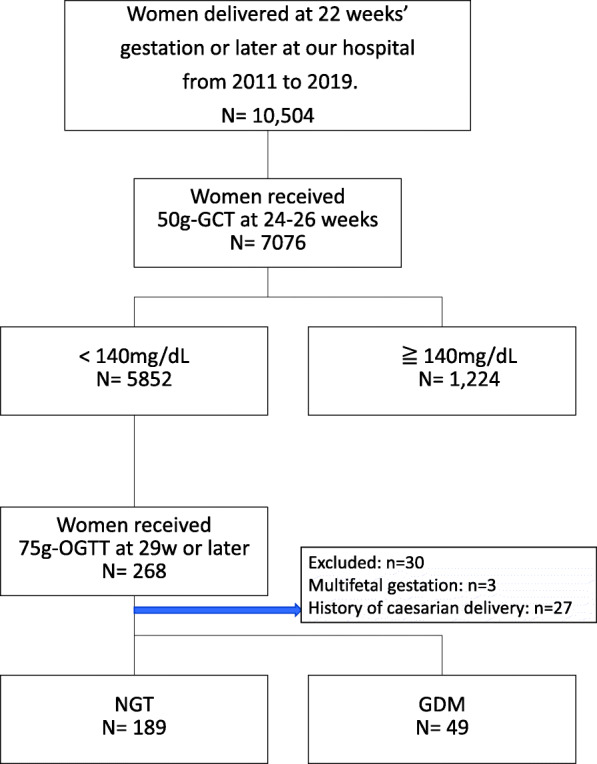
Flowchart of subjects selection

The total number of deliveries after 22 weeks of gestation at our hospital during the study period was 10,548. Of these, 7076 patients underwent the 50-g GCT, and 5852 had normal results (< 140 mg/dL). Among them, 268 patients underwent the 75-g OGTT after 29 weeks of gestation. Of them, 238 patients were included in the analysis, excluding 3 women with multiple gestations and 27 who had a previous caesarean delivery. Among these subjects, 189 and 49 women were in the NGT group and the GDM group, respectively (Fig. 1).

Table 1 summarises the maternal backgrounds of the two groups. The median ages at delivery were 32 years (IQR, 28–35 years) and 31 years (IQR, 28–35 years) in the NGT group and GDM group, respectively, with no significant difference observed between groups (*p* = 0.46). There were no differences in height, pre-pregnancy weight, and BMI. Weight at delivery was lesser in the GDM group than in the NGT group (60.6 kg [IQR, 56.5–69.9] and 64.6 kg [IQR, 58.6–71.1], respectively); however, the difference was not statistically significant (*p* = 0.14). Gestational weight gain was significantly lower in the GDM group than in the NGT group (9.0 kg and 11.0 kg, respectively; *p* = 0.02). There was a higher proportion of male newborns in the GDM group than in the NGT group (71.4 and 51.8%, respectively; *p* = 0.01). Three women (6.1%) in the GDM group were treated with insulin.

**Table 1:** Characteristics of each group

	NGT *n* = 189	GDM *n* = 49	*p*-value
Maternal age (yo)	32 (28–35)	31 (28–35)	0.46
Maternal height (cm)	159 (155–162)	157 (153–163)	0.16
Pre-pregnancy weight (kg)	52.8 (48.0–58.0)	50.6 (47.0–57.5)	0.47
Pre-pregnancy BMI (kg/m2)	20.7 (19.2–22.5)	20.5 (18.8–22.8)	0.90
Weight at delivery (kg)	64.6 (58.6–71.1)	60.6 (56.5–69.9)	0.14
BMI at delivery (kg/m2)	25.4 (23.5–27.6)	24.9 (22.4–27.8)	0.48
Gestational weight gain (kg)	11.0 (8.9–13.8)	9.0 (7.2–12.8)	0.02
Multipara	72 (38.1)	20 (40.8)	0.73
Male newborns	98 (51.8)	35 (71.4)	0.01
Insulin treatment	0 (0)	3 (6.1)	–

Data are expressed as n (%) or median (*IQR* Inter Quartile Range)

Table 2 summarises the gestational and neonatal outcomes of both groups. The rates of diabetes-related complications were 13.8 and 18.4% in the NGT group and GDM group, respectively; this difference was not statistically significant (aOR, 1.31; 95% CI, 0.56–3.06; *p* = 0.53). No statistically significant differences in the rates of macrosomia, shoulder dystocia, hypoglycaemia, or hyperbilirubinemia were observed between the two groups. None of the infants developed RDS. The overall caesarean delivery rate was significantly higher in the GDM group than in the NGT group (28.6 and 15.9%, respectively; aOR, 2.41; 95% CI, 1.07–5.43; *p* = 0.04). Similarly, the rate of elective caesarean deliveries was higher in the GDM group than in the NGT group (16.3 and 5.3%, respectively; aOR, 3.60; 95% CI, 1.27–10.19; *p* = 0.01). However, the rates of emergency caesarean deliveries were not significantly different between groups (10.6% for the GDM group and 12.2% for the NGT group; aOR, 1.25; 95% CI, 0.43–3.64; *p* = 0.74). The median birth weights were 3222 g (IQR, 2911–3541 g) in the NGT group and 3189 g (IQR, 2949–3496 g) in the GDM group; however, no significant difference was observed between both groups (aRC, − 13.3; 95% CI, − 83–56; *p* = 0.71). The rates of LGA status were 31.6% in the NGT group and 24.0% in the GDM group, which were higher than the general rates; however, no significant difference was observed between groups (*p* = 0.20). The rates of SGA status were lower than the general rates of 5.2% in the NGT group and 6.0% in the GDM group; however, the difference was not significant. Additionally, no significant differences in low APS, low UApH, and NICU admission rates were observed between groups.

**Table 2:** Pregnancy delivery and neonatal outcomes

	NGT	GDM			*P* value
	*n* = 189	*n* = 49	^a^aOR	95% CI	
Diabetes-related composite complication^b^	26 (13.8)	9 (18.4)	1.31	0.56–3.06	0.53
Macrosomia	10 (5.3)	3 (6.1)	0.98	0.25–3.86	0.97
Shoulder dystocia	6 (3.2)	3 (6.1)	1.6	0.37–6.85	0.53
Hypoglycemia	2 (1.1)	2 (1.1)	3.71	0.49–27.84	0.2
Hyperbilirubinemia	11 (5.8)	2 (4.1)	0.74	0.15–3.61	0.71
RDS	0	0	–	–	–
Preeclampsia	11 (5.8)	0	–	–	–
Total caesarian delivery	30 (15.9)	14 (28.6)	2.41	1.07–5.43	0.03
Elective caesarian delivery	10 (5.3)	8 (16.3)	3.6	1.27–10.19	0.02
Emergency caesarian delivery	20 (10.6)	6 (12.2)	1.25	0.43–3.64	0.68
Instrumental delivery	17 (9.0)	3 (6.1)	0.69	0.18–2.59	0.58
Large for gestational age	59 (31.2)	11 (22.5)	0.54	0.25–1.17	0.12
Small for gestational age	11 (5.8)	3 (6.1)	1.01	0.26–3.90	0.99
Apgar score at 5 min < 7	0	0	–	–	–
UApH < 7.1	3 (1.6)	2 (4.1)	2.55	0.39–16.50	0.33
NICU admission	9 (4.8)	4 (8.2)	1.68	0.48–5.86	0.41
Neonatal admission	17 (9.0)	5 (10.2)	1.07	0.37–3.15	0.9
			^a^aRC		
Neonatal weight (g)	3222 (2911–3541)	3182 (2949–3496)	−24	−97-49	0.52
Gestational age at delivery (weeks)	39.6 (38.7–40.6)	39.6 (38.7–40.4)	0.04	−0.16-0.24	0.69

Data are expressed as median (IQR) or n (%)

^a^*aOR/ aRC* Adjusted Odds ratio/ regression coefficient, adjusted for maternal age, non-pregnant BMI, childbirth experience, and sex of the infant

^b^Diabetes-related composite complication was defined as any one of the following: macrosomia, shoulder dystocia, neonatal hypoglycaemia, neonatal hyperbilirubinemia, or RDS

## Discussion

This study showed that the frequency of diabetes-related composite complications for women diagnosed with GDM during the third trimester did not differ from that of pregnant women with NGT. More cases of elective caesarean deliveries were observed in the GDM group than in the NGT group; however, the rates of emergency caesarean deliveries were not significantly different between groups.

In this study, the rates of diabetes-related composite complications did not differ between women with GDM diagnosed and treated during the third trimester and women with NGT. However, diagnosing GDM and initiating interventions during the later stages of pregnancy may be too late to improve delivery outcomes. Limited reports have examined the effectiveness of diagnosing GDM and initiating interventions during late pregnancy. Arbib et al. reported a single-centre, retrospective study of pregnant women who underwent the 100-g 3-h OGTT during the third trimester despite normal 50-g GCT results. They reported that the group diagnosed with and treated for GDM during the third trimester birthed infants with lower birth weights than the NGT group; however, no difference in caesarean delivery rates or other outcomes was observed between groups [14]; the previous study results contradicted those of the present study. Differences in diagnostic criteria may have significantly contributed to this. Diagnosing and treating GDM after 29 weeks of gestation using the IADPSG criteria did not appear effective in this study.

It remains unknown whether the IADPSG criteria developed based on the results of the HAPO study of pregnant women between 24 and 32 weeks of gestation can be applied to different weeks of gestation to correctly detect abnormal glucose tolerance or pregnant women at high risk. Furthermore, OGTT results have been reported to vary according to the weeks of gestation [15, 16].

This study showed that more elective caesarean deliveries were performed for the GDM group than the NGT group. In Japan, GDM with an estimated foetal weight of more than 4000 g are indications for allowing patients to choose a scheduled caesarean delivery to avoid shoulder dystocia [17]. At our institution, we follow this policy and perform elective caesarean deliveries at the request of the patients. We speculated that a diagnosis of GDM also allows clinicians to select caesarean delivery to avoid shoulder dystocia because our data showed higher elective caesarean delivery rates but equivalent emergency caesarean delivery rates in the GDM group. Increased caesarean delivery rates for pregnant women diagnosed with GDM during late pregnancy have been reported by a previous study by Sasson et al., who reported that in a retrospective study of pregnant women who underwent the 100-g 3-h OGTT after 37 weeks of gestation despite normal GCT results during mid-pregnancy, there was a higher caesarean delivery rate in the GDM group; however, other pregnancy delivery outcomes were unchanged [18]. Fonseca et al. reported a prospective study of the 75-g 2-h OGTT performed at 32–36 weeks of gestation for pregnant women with normal glucose tolerance screening results during mid-pregnancy (with results withheld) to observe differences in delivery outcomes [19]. Forty-five of 334 pregnant women (13.5%) had abnormal OGTT results at 32–36 weeks of gestation, and they had more induced deliveries and caesarean deliveries than women with normal results. However, there were no differences in child size or neonatal outcomes. The results of this previous study are consistent with those of the present study because it used the IADPSG criteria and higher caesarean delivery rates were observed in the abnormal OGTT group; however, they differ from those of the current study because the results were blinded and the group with abnormal values was not treated.

This is the first study to examine the delivery outcomes of pregnant women diagnosed with and treated for GDM after 29 weeks of gestation using the same thresholds as the IADPSG criteria.

This study had several limitations. First, selection bias may have existed in this study because it was a single-centre, retrospective study with small sample size. Second, the long-term prognoses of both mothers and children have not been studied. Pregnant women with GDM are at increased risk for metabolic syndrome [20], type 2 diabetes [21], and cardiovascular disease [22]. Similarly, GDM is considered to affect future metabolic abnormalities and neurodevelopmental prognoses of children [23–26].

## Conclusion

In this study, the rates of diabetes-related composite complications of pregnant women diagnosed with GDM during the third trimester were not different from those of pregnant women with NGT. However, the rate of elective caesarean delivery was higher in the group diagnosed with GDM during late pregnancy than in the NGT group. These results may have been influenced by the fact that pregnant women with GDM and suspected LGA foetuses were given the option of elective caesarean delivery. Further studies of the significance of diagnosing and treating GDM during late pregnancy are warranted.

## Data Availability

The datasets used and/or analysed during the current study are available from the corresponding author on reasonable request.

## Abbreviations

IADPSG: International Association of Diabetes and Pregnancy Study Group
GDM: Gestational Diabetes Mellitus,
OGTT: Oral Glucose Tolerance Test
WHO: World Health Organization
GCT: Glucose Challenge Test
NGT: Normal Glucose Tolerance
OR: Odds Ratio
RC: Regression Coefficient
aOR: Adjusted Odds Ratio.
aRC: Adjusted Regression Coefficient
CI: Confidence Interval
LGA: Large for Gestational Age
SGA: Small for Gestational Age
HAPO: Hyperglycaemia and Adverse Pregnancy Outcome
JSOG: Japan Society of Obstetrics and Gynecology
BMI: Body Mass Index
GWG: Gestational Weight Gain
RDS: Respiratory Distress Syndrome
UA: Umbilical artery
APS: Apgar score
NICU: Neonatal Intensive Care Unit
